# Prostatic artery embolization and imaging-based biomarkers: current tools in monitoring treatment response and emerging strategies

**DOI:** 10.1007/s00261-026-05628-5

**Published:** 2026-06-26

**Authors:** Gavin Wu, Umairullah Lodhi, Robert Tenney, Mina S. Makary, Jason C. Hoffmann

**Affiliations:** 1 The Ohio State University Wexner Medical Center, Columbus, USA; 2 NYU Langone Hospital - Long Island, Mineola, USA

**Keywords:** Prostate artery embolization, Benign prostatic hyperplasia, Imaging biomarkers, Treatment response, Magnetic resonance imaging, Lower urinary tract symptoms

## Abstract

Prostatic artery embolization (PAE) is a safe and effective minimally invasive treatment for lower urinary tract symptoms (LUTS) attributed to benign prostatic hyperplasia (BPH), including in patients with large prostates, who are poor surgical candidates or wish to avoid the morbidity of conventional surgery or transurethral interventions. As PAE becomes more widely adopted for BPH-related LUTS, objective measures of assessing treatment response are needed beyond symptom-based tools such as the International Prostate Symptom Score (IPSS) and Quality-of-Life (QoL) index. Imaging biomarkers complement these subjective endpoints by quantifying structural, functional, ischemic, and microstructural changes within the prostate before and after embolization. This review summarizes the principal imaging modalities used for biomarker assessment in PAE, with emphasis on ultrasound, magnetic resonance imaging (MRI), and computed tomography (CT) scans, and examines the current literature on imaging biomarkers of treatment response. Volumetric biomarkers, including whole prostate volume, central gland volume, and zonal volumetry index, provide quantitative assessment of gland reduction and may help predict clinical improvement, with zonal parameters showing stronger associations with symptomatic outcomes than whole-gland measurements alone. Functional biomarkers such as postvoid residual volume offer objective evaluation of bladder emptying alongside volumetric assessment, whereas ischemia-based biomarkers derived from contrast-enhanced MRI directly reflect embolization-induced devascularization. Diffusion-weighted imaging and apparent diffusion coefficient mapping provide additional information regarding post-embolization tissue remodeling, with b-value selection influencing early infarct detection. Emerging tools, including ultrasound elastography, radiomics, and artificial intelligence-assisted image analysis, may further refine patient selection, standardize response assessment, and improve predictive modeling, though prospective PAE-specific validation remains needed. Imaging biomarkers provide a more objective framework for evaluating PAE treatment response, with the potential to standardize outcome reporting, facilitate earlier identification of treatment success or failure, and guide biomarker-driven clinical decision-making. Prospective validation in larger PAE-specific cohorts remains essential before broader clinical adoption.

## Background

Benign prostatic hyperplasia (BPH) is a condition defined by the nonmalignant proliferation of stromal and epithelial tissue within the transitional zone of the prostate [1]. BPH prevalence increases markedly with age, with more than 80% of men older than 70 affected [2]. As the global population ages, the number of symptomatic cases is expected to rise [3].

BPH commonly presents as lower urinary tract symptoms (LUTS), including urinary frequency, urgency, decreased strength of urinary stream, nocturia, and incomplete bladder emptying [4]. First-line management consists of lifestyle modification and pharmacologic therapy with α-blockers or 5α-reductase inhibitors, which work in tandem to address both the dynamic and static components of prostatic obstruction [1, 2]. Nonetheless, medical therapy often produces modest symptom relief and may be associated with adverse effects including orthostatic hypotension, loss of libido, and erectile dysfunction [3]. Some patients are unable to tolerate medical therapy or continue to progress despite treatment, warranting more invasive management [2, 3].

Surgical options such as transurethral resection of the prostate (TURP) and open prostatectomy, while effective, carry considerable morbidity including erectile dysfunction, long-term ejaculatory dysfunction, urinary tract infections, and incontinence [1, 3]. Prostatic artery embolization (PAE) has emerged as a minimally invasive alternative in which selective catheterization and occlusion of the prostatic arteries induce ischemic volume reduction of the gland and improvement of clinical symptoms [1–3, 5]. In a large meta-analysis of 13 PAE studies totaling 1,254 patients, mean International Prostate Symptom Score (IPSS) improvement at one year was 16.2 points, quality-of-life (QoL) improvement was 3.0 points, and mean prostate volume decreased by 26%, with results remaining statistically significant at three years [3]. In addition, compared to conventional surgical approaches, PAE does not require general anesthesia or inpatient hospitalization, and carries a lower risk of sexual health side effects such as retrograde ejaculation and erectile dysfunction [6].

Post-procedural monitoring in PAE has traditionally relied on the IPSS, a validated questionnaire that provides a subjective measure of urinary symptom severity on a 35-point scale [3, 6]. While symptom scores certainly are important, imaging biomarkers may offer a more direct, anatomy-based complement to symptom scores for evaluating PAE treatment response. For example, magnetic resonance imaging (MRI)-derived measurements, such as central gland volume (CGV) and whole prostate volume (WPV), have demonstrated strong correlations with clinical outcomes following PAE [7]. Pre- and post-procedural imaging biomarkers may therefore provide an objective framework for treatment assessment, encompassing baseline gland characterization, response evaluation, and follow-up planning [1]. This review was based on a targeted literature search of PubMed for English-language studies on prostatic artery embolization and imaging biomarkers published through May 2026, with emphasis on studies evaluating structural, functional, ischemic, and diffusion-based imaging changes after embolization.

## Imaging modalities for biomarker assessment

Imaging biomarkers of PAE treatment response are primarily assessed and obtained through ultrasound (US), MRI, and computed tomography (CT) scans. US provides accessible, radiation-free assessment of prostate volume and bladder function, while multiparametric MRI offers the most comprehensive soft-tissue characterization for zonal anatomy, perfusion, and diffusion-based tissue analysis. The key features of each modality, including their role in biomarker assessment, advantages, and limitations, are summarized in Table 1.

### Ultrasound

US is a commonly utilized imaging modality in PAE because it is widely available, free of ionizing radiation, and relatively inexpensive. Many patients presenting for PAE consultation will have undergone prostate US as part of their prior urologic evaluation [6]. Prostate volume can be measured with either transabdominal or transrectal US. Transrectal US generally provides greater accuracy, particularly for glands smaller than 50 mL, because of its higher-resolution and closer proximity to the gland [1]. Postvoid residual urine volume (PVR) may also be measured with US and serves as a biomarker of urinary function in the setting of bladder outlet obstruction [2]. The Society of Interventional Radiology (SIR) currently recommends PVR as a principal noninvasive measure for pre-procedural evaluation [8]. Its role as a biomarker is discussed further in Sect. 3.2.

Contrast-enhanced ultrasound (CEUS) has also been described as an adjunctive modality in PAE. For instance, intra-arterial CEUS has been used intra-procedurally to confirm target vessel access and embolization while identifying extra-prostatic perfusion, potentially reducing reliance on cone-beam CT and associated radiation exposure [9]. Intravenous CEUS has also been explored for post-procedural assessment, with transabdominal CEUS demonstrating reproducible evaluation of non-enhancing prostatic tissue after embolization [9]. However, current evidence is limited to small case series, with no consistent correlation with clinical outcomes [9, 10].

US has several limitations as a biomarker imaging tool. It is operator dependent and therefore subject to inter-observer variability [1, 9]. It also has limited ability in distinguishing prostate zones, characterizing infarction, or evaluating pelvic vasculature. In addition, image quality may be reduced in patients with obesity, very large prostates, or inadequate bladder filling [1].

### MRI

MRI is particularly well suited for measurement of treatment response in PAE because it provides comprehensive evaluation of pre-procedural zonal anatomy and post-treatment tissue changes [11]. Its multisequence capability allows it to quantify volumetric, ischemia-based, and diffusion-based biomarkers. For example, T2-weighted imaging delineates prostatic zonal anatomy and supports measurement of WPV and CGV [1]. Dynamic contrast-enhanced (DCE) sequences, acquired with T1-weighted imaging, characterize tissue perfusion and provide the basis for ischemia-based biomarker assessment after embolization [12, 13]. Diffusion-weighted imaging (DWI) and apparent diffusion coefficient (ADC) mapping characterize water mobility within the treated gland and provide complementary information about microstructural changes. Notably, b-value selection may influence the sensitivity of infarct detection in the post-procedural setting [14]. Its roles in volumetric, ischemia-based, and diffusion-based biomarker assessment are discussed further in Sect. 3.1, 3.3, and 3.4.

### CT

CT has a more limited role than US or MRI in treatment response assessment in PAE. It is primarily used in pre-procedural evaluation of PAE candidates and intra-procedural guidance, with CT angiography favored by many operators for its speed, spatial resolution, and ability to depict arterial anatomy [6]. However, when post-procedural MRI cannot be performed, contrast-enhanced CT may provide structural confirmation of embolization and volumetric evaluation. Non-enhancing regions on post-contrast imaging correspond to infarct zones, and advanced perfusion CT techniques measuring blood flow, blood volume, and mean transit time may help characterize devascularization in patients [1]. It is important to note that CT has inferior soft-tissue contrast compared to MRI, which limits its ability to discriminate prostatic zones, characterize detailed infarct morphology, or provide diffusion-based analysis [1].

**Table 1:** Imaging modalities for biomarker assessment in PAE

Modality	Role	Biomarkers assessed	Strengths	Limitations
Ultrasound (US)	Commonly used to measure prostate volume and assess bladder function; suitable for serial follow-up; CEUS may also provide assessment of post-procedural ischemia	Whole prostate volume (WPV); postvoid residual volume (PVR)	Widely available; low cost; no ionizing radiation; no contrast required	Operator-dependent; limited zonal discrimination; reduced image quality in patients with obesity, very large glands, or inadequate bladder filling
Magnetic resonance imaging (MRI)	Excellent assessment of prostatic zonal anatomy, perfusion, and microstructural changes	WPV; central gland volume (CGV); zonal volumetry index (ZVi); imaging evidence of ischemia and infarction; diffusion-weighted imaging (DWI)/apparent diffusion coefficient (ADC) mapping	Superior soft-tissue contrast; multisequence capability; no ionizing radiation	Higher cost; less accessible than US; longer acquisition times; gadolinium required for some sequences; contraindicated in some patients
Computed tomography (CT)	Adjunctive modality in biomarker assessment; primary role in current clinical practice involves pre-procedural vascular mapping and intra-procedural guidance with CT angiography	WPV; imaging evidence of post-embolization infarction	High spatial resolution; quick acquisition times	Limited zonal discrimination and inferior soft-tissue contrast; ionizing radiation

## Imaging biomarkers of treatment response

The imaging biomarkers for treatment response in PAE can be grouped into four broad categories: volumetric, functional, ischemia-based, and diffusion-based. Each reflects a different aspect of post-treatment response, including gland reduction, bladder emptying, embolization-induced devascularization, and microstructural tissue changes. This framework provides a practical basis for objective response evaluation in PAE and is summarized in Table 2.

### Volumetric biomarkers

Volumetric biomarkers are central to imaging-based assessment in PAE because they provide quantification of baseline gland morphology and structural changes following embolization. In addition to capturing post-procedural gland shrinkage, volumetric biomarkers may identify baseline anatomic features that predict favorable clinical outcomes. For this reason, zonal volumes, particularly of the central gland, have emerged as a more anatomically specific approach to volumetric treatment response assessment [1, 7].

#### Whole prostate volume

**Fig. 1:**
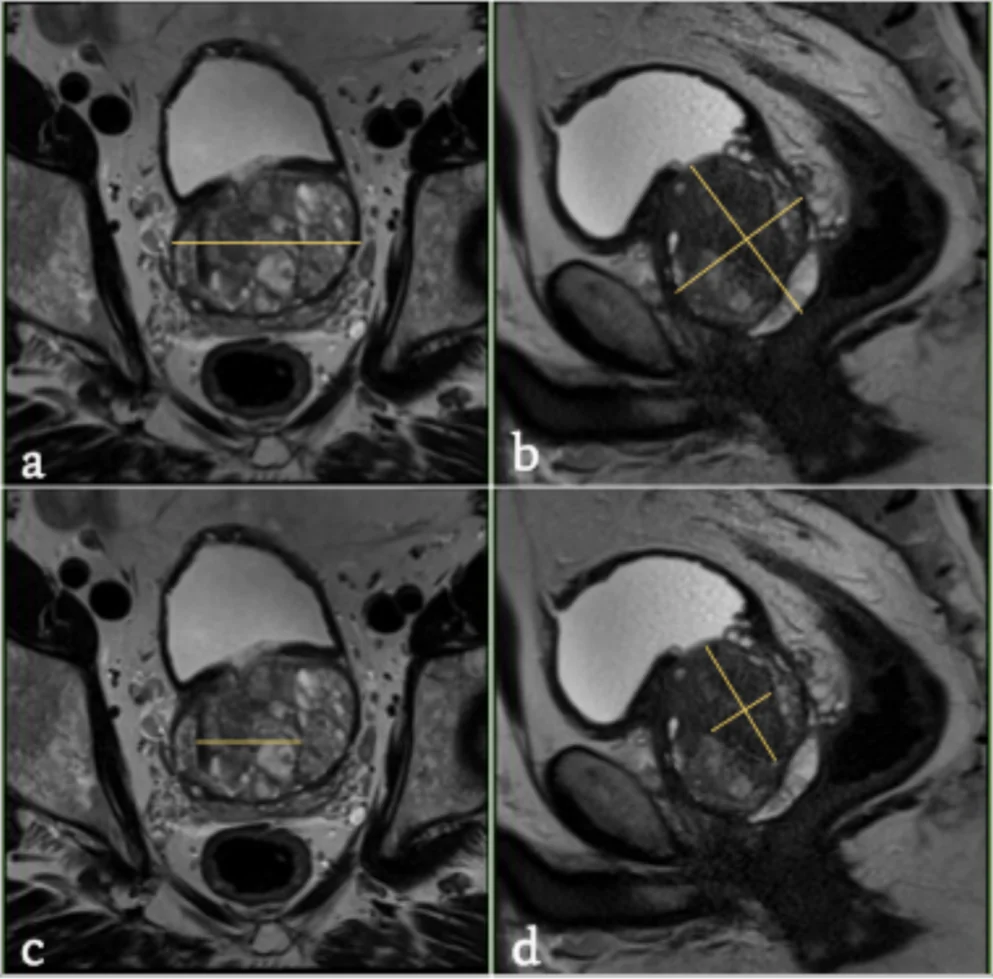
Volumetric biomarker assessment prior to PAE. **a** Axial and (**b**) sagittal T2-weighted MRI demonstrate measurements used to calculate whole prostate volume (WPV). **c** Axial and (**d**) sagittal T2-weighted images demonstrate measurements of the central gland used to calculate central gland volume (CGV), from which the zonal volumetry index (ZVi = CGV/WPV) may also be derived

WPV is a common image-derived biomarker in PAE that provides a direct measure of gland size before and after treatment. It can be obtained with either US, CT, or MRI using the ellipsoid formula (width × height × length ×0.52) or with volumetric software. Although US is frequently used given its accessibility and lack of ionizing radiation, MRI provides superior soft-tissue characterization and is particularly useful for pre-procedural zonal anatomy assessment and post-procedural evaluation of infarction (Fig. 1a and b) [1, 8].

Reductions in WPV after embolization are generally interpreted as a reflection of treatment-related devascularization and gland volume reduction. In a retrospective study of 93 patients undergoing PAE for LUTS secondary to BPH, de Assis et al. reported a mean WPV reduction of 30.6% at 6 months, with larger baseline WPV significantly correlating with QoL improvement [7].

While WPV provides useful information on gland reduction, its association with clinical improvement has not been consistent across studies [4, 7]. For instance, in the de Assis et al. cohort, baseline WPV correlated significantly with QoL improvement but not IPSS, suggesting that whole-gland size alone may not fully reflect the tissue burden most relevant to symptom response [7]. This may be because WPV includes peripheral zone tissue, which is not the primary target of embolization, and because factors such as detrusor dysfunction may drive symptoms independently of gland volume [1, 15].

#### Central gland volume and zonal volumetry index

The central gland, which includes the transitional and central zones, is the main site of BPH-related hyperplastic growth and the principal target of PAE. CGV may therefore serve as a more targeted volumetric biomarker of clinical response, reflecting the hyperplastic tissue most directly implicated in bladder outlet obstruction [1, 7, 16]. In a prospective study by Ali et al., CGV decreased in 100% of patients at 6 months, with a median reduction of 26.7%, compared with a median WPV reduction of 18.2% [17]. Because of its superior soft-tissue resolution and zonal discrimination, MRI is the preferred modality for measuring CGV (Fig. 1c and d) [1].

CGV has also shown a stronger correlation with clinical outcomes than WPV. For instance, in the study led by de Assis et al., higher baseline CGV correlated significantly with greater improvement in both IPSS and QoL, whereas baseline WPV correlated significantly with QoL improvement only. Greater CGV reduction was also directly associated with improvement in both IPSS and QoL at 6-month follow-up [7].

De Assis et al. also evaluated the zonal volumetry index (ZVi) in PAE. While CGV reflects the absolute burden of hyperplastic tissue associated with outlet obstruction, ZVi (ZVi = CGV/WPV) measures the proportion of total gland volume occupied by the central gland and may therefore better approximate the fraction of BPH-related tissue amenable to embolization [7]. In their cohort, higher baseline ZVi was significantly associated with greater clinical improvement, and a ZVi threshold greater than 0.45 predicted favorable clinical outcomes with 85% sensitivity and 75% specificity [7].

While CGV and ZVi show promise, their correlation with symptomatic outcomes has not been consistent across studies. For instance, Lin et al. reported significant decreases in WPV and CGV after PAE, with larger reductions observed in clinically successful patients than in failures [4]. However, no significant linear correlation was identified between zonal volume changes and IPSS or QoL, highlighting the need for prospective validation before these biomarkers can be considered standardized response markers [4].

### Functional biomarkers

Functional biomarkers assess pre- and post-procedural bladder emptying and urinary outflow, providing objective insight into the physiologic consequences of outlet obstruction. In PAE, PVR is recommended by the SIR as an objective measure for pre-procedural evaluation [8]. Because LUTS may persist despite prostate volume reduction, particularly in cases where bladder dysfunction is related to chronic obstruction, PVR may provide objective functional information that volumetric biomarkers alone cannot capture [18].

#### Postvoid residual volume

**Fig. 2:**
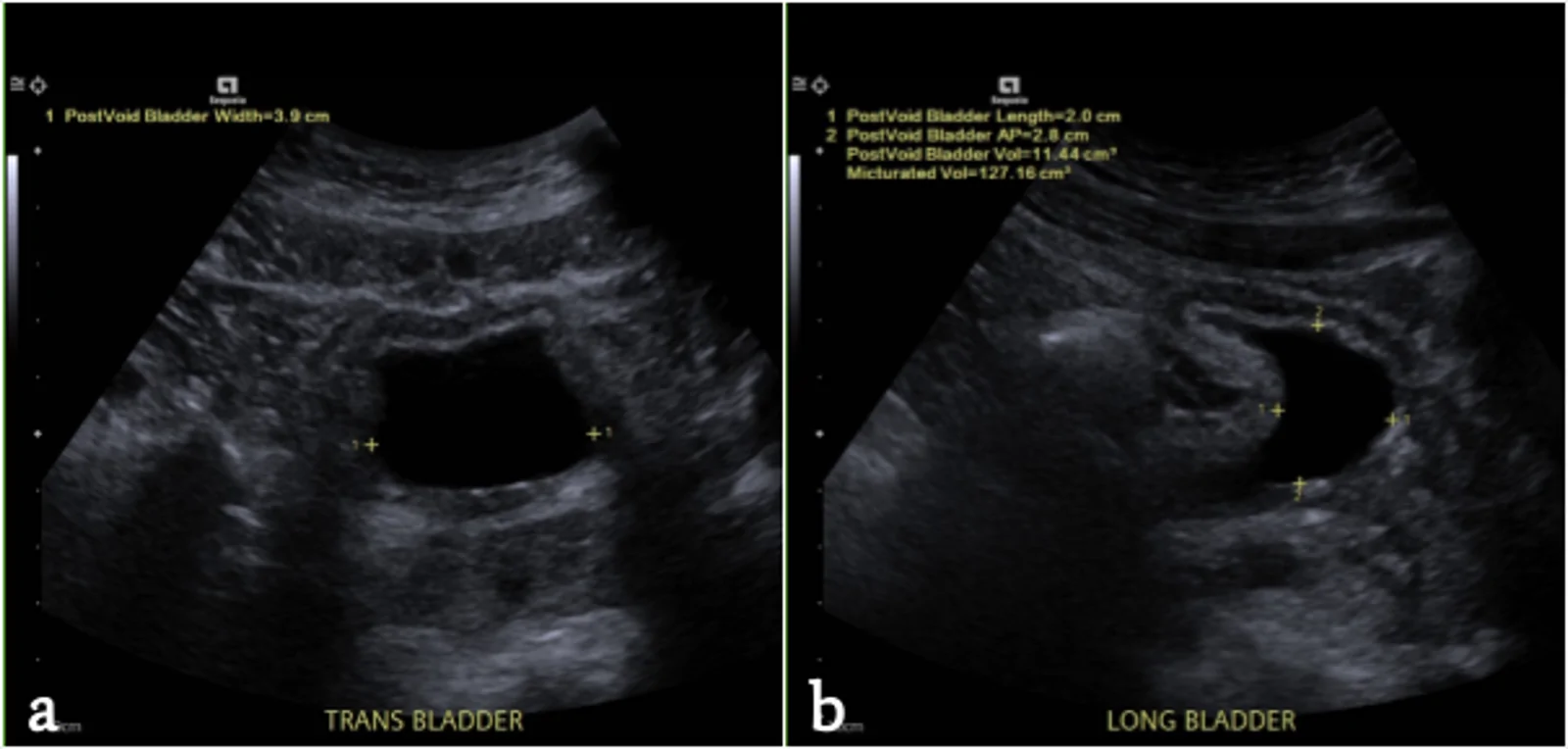
Measurement of postvoid residual volume (PVR). **a** Transverse and (**b**) longitudinal transabdominal ultrasound images obtained after voiding demonstrate bladder measurements used to calculate PVR using the ellipsoid formula (width × height × length ×0.52)

PVR reflects incomplete bladder emptying in the setting of prostatic resistance and serves as a measure of bladder outlet obstruction. It is obtained with either transabdominal or transrectal US and calculated using the ellipsoid formula (PVR = width × height × length ×0.52) (Fig. 2). PVR quantifies the severity of baseline functional obstruction alongside structural parameters such as prostate volume [1, 8]. The SIR Reporting Standards identify both PVR and peak urinary flow rate (Qmax) as required noninvasive objective measures for pre-procedural assessment in PAE studies, noting their value in both initial diagnostic evaluation and assessment of treatment response [8].

Nonetheless, reported changes in PVR after PAE have not been uniform across cohorts, despite many studies finding significant improvements in PVR after PAE. In a large retrospective study of 186 patients, Bilhim et al. reported a statistically significant overall improvement in PVR after PAE, with the greatest mean reduction occurring at 6 months (−45.3 mL) [19]. In contrast, Lin et al. found no significant reduction in PVR despite significant improvements in IPSS and QoL (52.9 ± 36.8 mL pre-PAE vs. 39.7 ± 30.1 mL post-PAE) [4]. These findings suggest that while PVR may improve after PAE, its reduction does not consistently correlate with symptomatic outcomes, and it is therefore best interpreted as one component of post-PAE assessment rather than a standalone indicator of treatment success. This inconsistency across cohorts may be due to inter-observer variability, and Dias et al. recommend supplementing PVR with the residual fraction (residual fraction = [PVR ×100]/pre-void volume) to improve measurement reproducibility [1].

### Ischemia-based biomarkers

**Fig. 3:**
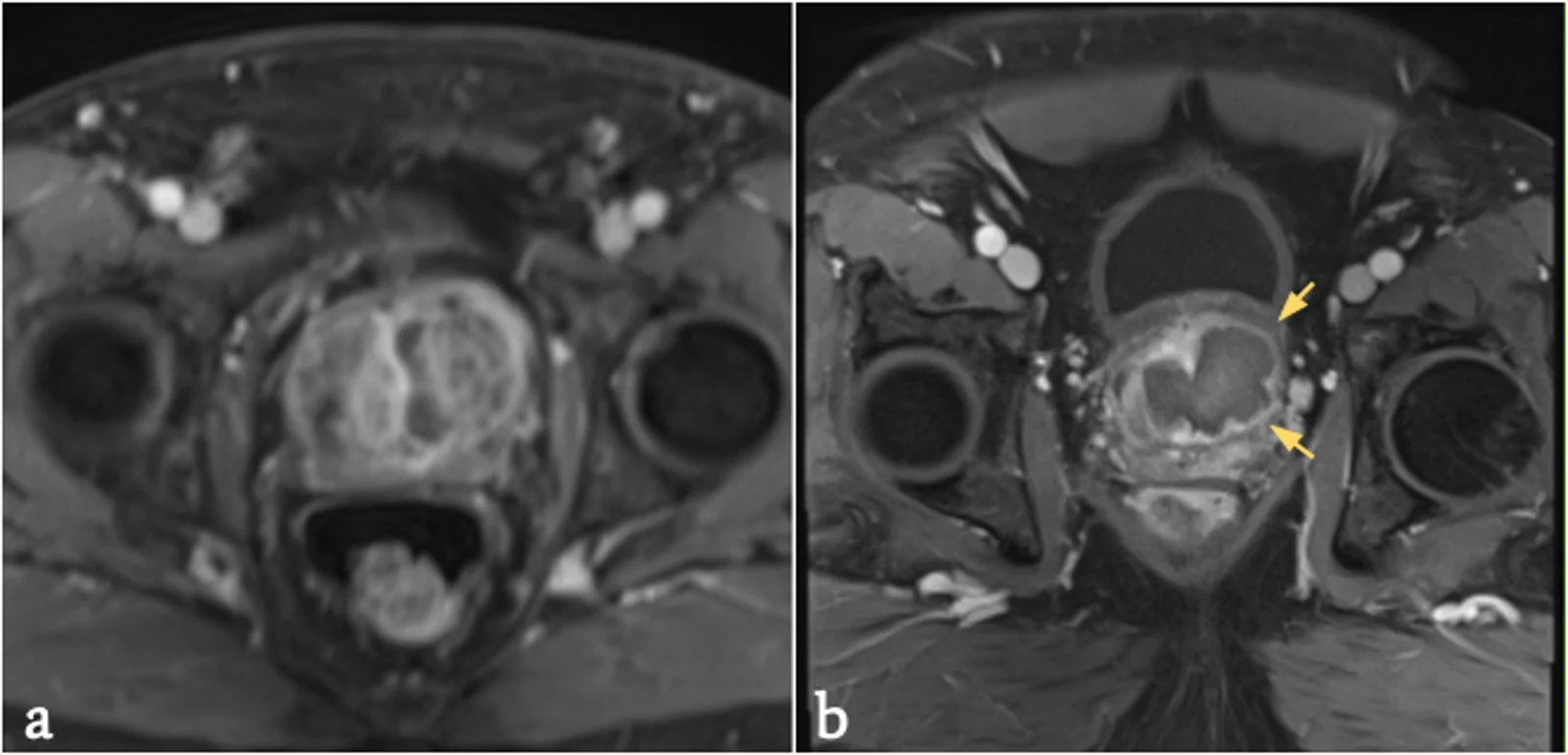
Ischemia-based biomarker assessment in PAE. **a** Pre-PAE axial contrast-enhanced T1-weighted MRI demonstrates heterogeneous enhancement of the enlarged central gland. **b** Contrast-enhanced T1-weighted MRI obtained one month after PAE in the same patient demonstrates newly developed non-enhancing areas within the central gland (arrows), consistent with embolization-induced ischemia and infarction. Typical post-PAE infarcts appear as non-enhancing regions on contrast-enhanced T1-weighted imaging, with corresponding signal changes on T2-weighted images

Ischemia-based biomarkers reflect embolization-induced devascularization within the treated gland and are typically identified on DCE-MRI as new non-enhancing tissue on post-gadolinium T1-weighted images (Fig. 3). Contrast-enhanced MRI is the primary modality for this purpose, as it can detect these non-enhancing infarction zones and characterize associated signal changes on T1- and T2-weighted sequences [1, 20]. These regions are usually confined to the central gland, which is the main site of BPH-related hyperplasia and encompasses the transitional and central zones [20].

In practice, post-PAE ischemia assessment is centered on the post-contrast phase most reliably demonstrating non-enhancing infarcted tissue relative to enhancing residual gland [1, 12, 14]. For instance, delayed post-gadolinium T1-weighted images obtained at approximately 5 min post-contrast have been used to delineate non-enhancing infarction zones, though timing protocols remain heterogeneous across studies [21].

Evidence from several studies suggests a close relationship between post-PAE infarction and subsequent prostate volume reduction. Frenk et al. reported significant prostate volume reduction at 12 to 18 months in patients who developed infarcts (cumulative reduction 32.0% ± 19.2%, *p* < 0.001), while no significant reduction was observed in those without imaging-detected ischemia [20]. In another study, Lin et al. found that among patients in whom central gland infarction developed, reductions in WPV, CGV, and median lobe volume were each significantly larger than in patients without infarction (all comparisons *p* < 0.01) [4].

The degree of post-embolization ischemia has also been associated with improved clinical outcomes in PAE [12, 22]. In a cohort of 24 patients with acute urinary retention secondary to BPH, Kisilevzky et al. found that ischemic areas were identified in 87% of clinical successes compared with 11% of failures. Mean infarcted tissue volume was also substantially greater in clinical successes than failures (22% vs. 0.44% of gland volume, *p* = 0.0006). Of note, among all variables examined, percentage tissue ischemia was the strongest predictor of time to catheter removal, outperforming even prostate shrinkage rate [12].

While promising, ischemia-based biomarkers have not consistently predicted symptomatic improvement. Frenk et al. found no statistically significant association between infarct development and IPSS improvement [20]. Similarly, Lin et al. found no significant difference in clinical success rates between infarction-positive and infarction-negative patients (75% vs. 66.7%), despite significantly greater volumetric reductions in the former group [4].

Beyond inconsistencies in clinical correlation, the timing of post-procedural MRI remains an important consideration in interpreting ischemia-based biomarkers. Zhang et al. found that infarction is detectable on DCE-MRI as early as 10 days after PAE, while the greatest volumetric reduction occurred between baseline and 1 month, supporting one-month MRI as a practical early time point for routine outcome evaluation [14]. More recently, in a retrospective study of 63 patients, von Stempel et al. reported that infarction extent on three-month post-PAE MRI predicted 12-month clinical success, with infarction volume ≥ 5% yielding an AUC of 0.77, with 72% sensitivity and 80% specificity [21]. Taken together, these findings suggest that one-month MRI may be most useful for early ischemic and volumetric assessment, whereas three-month infarction extent may better reflect durability of response.

### Diffusion-based biomarkers

**Fig. 4:**
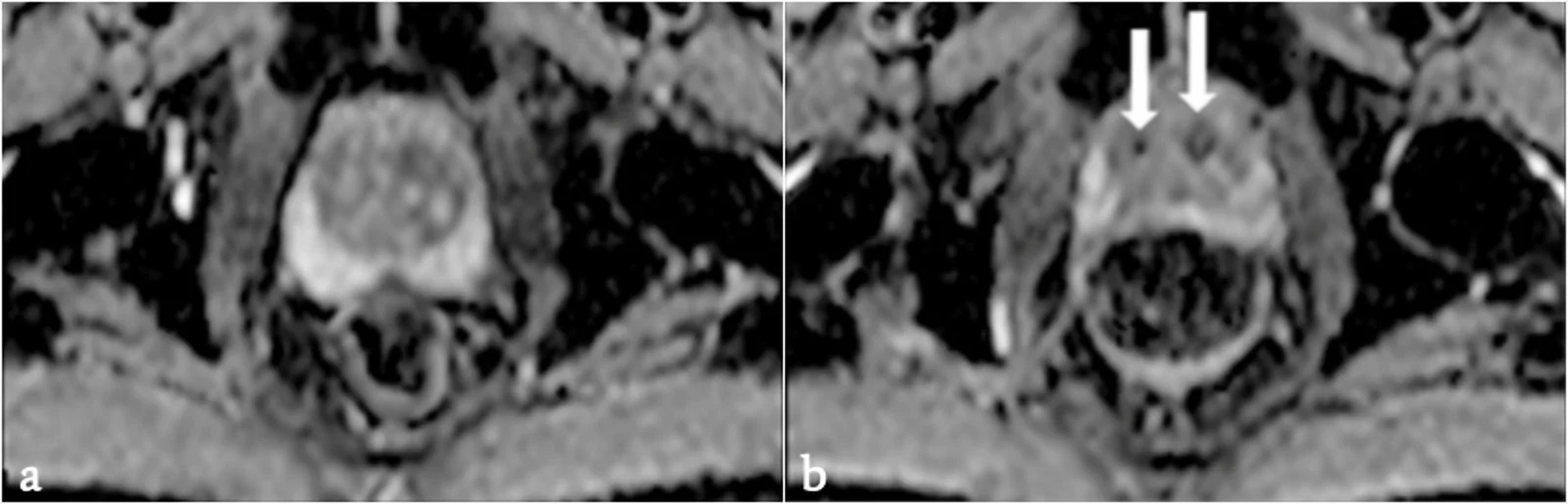
Diffusion-based biomarker assessment after PAE. **a** Axial diffusion-weighted image (DWI; b = 1000 s/mm²) of the prostate at baseline. **b** DWI at one month after PAE demonstrates signal changes within the central gland (arrows) reflecting decreased apparent diffusion coefficient (ADC) values, consistent with restricted diffusion in the setting of early post-embolization edema and infarction. Adapted from [16], licensed under CC BY

Diffusion-based biomarkers provide a different method of measuring treatment response in PAE by characterizing changes in water mobility within infarcted tissue. Unlike post-contrast sequences, which directly delineate non-enhancing ischemic zones, DWI and ADC mapping reflect microstructural changes within the treated gland (Fig. 4).

Current evidence suggests that DWI and ADC mapping provide insight into the evolution of post-embolization infarction over time. In a serial multiparametric MRI study of 28 patients, Zhang et al. evaluated diffusion changes at approximately 10 days and at 1, 3, 6, and 12 months post-PAE. At 10 days post-embolization, DWI at conventional b values of 1000 and 2000 s/mm² failed to characterize infarct extent, while imaging at b = 3000 s/mm² delineated the infarcted region [14]. This may reflect restriction of water molecule movement by early post-embolization edema and hemorrhage, which limits DWI sensitivity at conventional b values until resolution allows infarction to become apparent [14].

ADC values also appear to increase significantly after embolization, with the timing of significant change influenced by b-value selection. For instance, at b = 3000 s/mm², ADC values differed significantly from baseline at every post-embolization timepoint, whereas at b = 1000 and 2000 s/mm², significant differences were not observed until one month after embolization [14]. These findings suggest that DWI and ADC mapping are most informative when interpreted alongside contrast-enhanced ischemia assessment rather than as independent measures of treatment response. However, high b-value DWI at b = 3000 s/mm² is not proposed as a routine clinical protocol and remains investigational.

**Table 2:** Imaging biomarkers of treatment response after PAE

Biomarker	Modality	Measurement/Formula	Clinical significance	Reported clinical association	Limitations
Volumetric					
Whole prostate volume (WPV)	US, MRI	Ellipsoid formula = (width × height × length ×0.52); or measured with volumetric software	Measures overall gland size and structural response after PAE	Decreases after PAE; correlates with QoL in some cohorts	Does not consistently correlate with symptom improvement; not specific to BPH-related tissue burden
Central gland volume (CGV)	MRI	Ellipsoid formula applied to central gland (transitional and central zones) on T2-weighted imaging	Measures hyperplastic central gland burden most directly related to outlet obstruction	Decreases after PAE; stronger correlation with IPSS and QoL improvement than WPV	Zonal volume changes do not consistently correlate with IPSS on linear analysis; not yet standardized
Zonal volumetry index (ZVi)	MRI	ZVi = (CGV/WPV)	Measures contribution of central gland enlargement to total gland size	Decreases after PAE; ZVi > 0.45 predicts favorable outcomes (85% sensitivity, 75% specificity)	Limited supporting data; requires broader validation
Functional					
Postvoid residual volume (PVR)	US	Ellipsoid formula = (width × height × length ×0.52); measured immediately after voiding	Measures functional severity of bladder outlet obstruction	Decreases after PAE; provides objective functional follow-up alongside symptom scores	Operator-dependent; timing-sensitive; inconsistent correlation with symptom scores
Residual fraction	US	Residual fraction = ([PVR ×100]/pre-void volume)	Normalizes PVR to improve measurement reproducibility	Proposed to improve reproducibility of serial functional assessment	Limited PAE-specific data; requires prospective validation
Ischemia-based					
Imaging evidence of ischemia and infarction	MRI	Non-enhancing tissue on post-gadolinium T1-weighted imaging or DCE-MRI relative to baseline-enhancing tissue	Measures embolization-induced devascularization and infarction	Correlates with volumetric reduction; associated with clinical outcomes in select cohorts	Inconsistent correlation with IPSS; interpretation influenced by imaging timing (1–3 months post-PAE)
Diffusion					
DWI/ADC	MRI	ADC mapping from DWI at multiple b values (1000, 2000, 3000 s/mm²)	Measures tissue remodeling within infarcted prostate tissue	ADC decreases acutely after PAE and rises over time as infarction evolves; earliest changes detectable at b = 3000 s/mm²	Limited PAE-specific data; best interpreted with contrast-enhanced ischemia assessment
Emerging					
Shear Wave Velocity (SWV)/Elastic Modulus (EM)	US Elastography (US-E)	SWV (m/s); mathematically derived EM (kPa)	Measures tissue stiffness related to the dynamic component of outlet obstruction	Both decrease after PAE; baseline EM and SWV moderately correlate with 1-year IPSS; high baseline values predict suboptimal outcomes	Limited PAE-specific data; requires broader multicenter validation before routine clinical use

## Emerging tools and future directions

Emerging tools, including ultrasound elastography (US-E), radiomics, and artificial intelligence (AI)-based approaches, may refine treatment response assessment beyond the discussed imaging biomarkers. Although each approach remains investigational, they have the potential to improve patient selection, standardize response assessment, and reduce reliance on subjective endpoints.

### Ultrasound elastography

While the biomarkers previously discussed assess the static component of BPH, US-E is an emerging imaging tool that captures the dynamic component, defined as the increase in stromal smooth muscle tone contributing to prostatic stiffness and urinary resistance [2]. Decreased prostatic elasticity contributes to outlet obstruction independently of gland size, which may explain why prostate volume alone does not reliably predict symptom severity [2]. By measuring shear wave velocity (SWV) and the mathematically derived elastic modulus (EM), US-E quantifies these mechanical tissue properties, offering a distinct assessment of prostate function [23, 24].

Current evidence for US-E in PAE stems from two prospective studies by de Assis et al. In an initial pilot study of eight patients, US-E was performed using a transrectal approach, with mean SWV values obtained from the entire transitional zone bilaterally at the mid-gland level. A zone-specific stiffness ratio was derived by comparing 0.5 cm² region of interest (ROI) measurements at the periurethral transitional zone and adjacent peripheral zone [23]. At one month post-PAE, significant reductions in prostatic EM (47.24 to 33.14 kPa, −29.8%, *p* = 0.002) and SWV (4.63 to 3.75 m/s, −19.0%, *p* < 0.001) were observed, alongside a 45.36% reduction in the transitional-to-peripheral zone stiffness ratio [23].

A subsequent prospective study using a similar transrectal protocol, but with mean EM and SWV values derived from large elastographic maps encompassing the entire transitional zone rather than discrete ROI sampling, confirmed these findings [24]. At one month follow-up, de Assis et al. reported significant reduction in transitional zone stiffness, with mean SWV decreasing from 4.46 to 3.55 m/s (−20.0%, *p* < 0.0001) and EM decreasing from 46.3 to 34.4 kPa (−24.7%, *p* < 0.0001) [24]. These elastographic changes were also accompanied by significant improvements in IPSS, QoL, Qmax, prostate volume, and prostate-specific antigen (PSA) at three-month follow-up [24]. Of note, baseline elastographic parameters also showed moderate positive correlations with 1-year IPSS, suggesting a potential role for US-E in pretreatment patient selection (*R* = 0.62 for EM, *R* = 0.68 for SWV, *p* < 0.05 for both) [24].

US-E provides several advantages over other imaging modalities. It can be performed without contrast or ionizing radiation, allowing US-E to serve as an alternative to MRI in settings where MRI is unavailable or gadolinium is contraindicated [24]. In addition, serial elastographic assessment after PAE may provide a noninvasive means of detecting revascularization. Rising transitional zone stiffness or EM on follow-up imaging may indicate early revascularization before clinical symptoms recur, potentially prompting consideration of re-embolization [24]. Nonetheless, evidence supporting US-E in PAE remains limited, and broader validation will be needed before elastographic parameters can be incorporated as a routine biomarker.

### Radiomics/Machine learning

Radiomics refers to the high-throughput extraction of quantitative imaging features, including intensity, texture, and morphological descriptors, which are then used to build analytical models for outcome prediction and assessment of treatment response [25]. By quantifying tissue heterogeneity and other image-derived properties that might not be apparent on visual review, radiomics provides a novel quantitative framework for imaging-based assessment and clinical decision-making in PAE.

The majority of prostate radiomic studies to date have focused on prostate cancer rather than BPH. For example, multiparametric MRI-based radiomic models have been used to predict pathologic features, biochemical recurrence, and extracapsular extension in prostate cancer using T2-weighted, diffusion-weighted, and ADC-based MRI data [26]. As such, direct translation to the PAE setting is limited by differences in tissue biology, treatment mechanism, and imaging indication.

Nonetheless, post-PAE MRI captures features well-suited for computational analysis, including zonal volume changes and infarct extent. Machine-learning approaches may therefore help standardize quantitative post-PAE assessment through automated segmentation and biomarker integration. For instance, while manual ROI delineation incorporates expert knowledge, it is limited by inter- and intra-observer variability, and automated methods including U-net-based deep learning approaches may serve as a faster and more reproducible alternative [25]. In addition, machine learning models trained on radiomic signatures may eventually predict post-PAE outcomes, such as volumetric response or urinary flow recovery, from pre-procedural imaging alone [25, 26].

Several barriers currently limit clinical translation of radiomics and AI-based approaches in PAE. These include small sample sizes, variability in imaging acquisition protocols and analytic methodology, and an absence of large prospective PAE-specific validation studies [25, 26]. Addressing these limitations will require dedicated prospective PAE cohorts with standardized imaging protocols before radiomic and AI-derived biomarkers can be meaningfully incorporated into clinical practice.

### Clinical integration and future implications

The imaging biomarkers reviewed here are likely to be most useful when applied within a complementary, multiparametric framework rather than as isolated measurements. In practice, baseline MRI may provide the most complete pre-procedural characterization, with CGV and ZVi helping identify patients with a high burden of hyperplastic central gland tissue that may be more amenable to embolization. After treatment, contrast-enhanced MRI may help characterize the degree and distribution of post-embolization infarction, while adjunctive tools such as US-E may provide additional functional information in selected settings. Used together, these biomarkers may support patient counseling, guide follow-up planning, and help identify patients with incomplete treatment response who may require closer monitoring or repeat embolization. Although such a workflow has not yet been prospectively validated, it highlights how imaging biomarkers may eventually support more clinically actionable assessment after PAE and more standardized outcome reporting across institutions.

## Conclusion

PAE has emerged as a safe and effective minimally invasive treatment for BPH-related LUTS, with a favorable complication profile relative to conventional surgical options while also providing positive clinical outcomes in a majority of patients. Objective assessment of treatment response is vital because symptom scores alone do not fully capture the anatomic and physiologic changes that follow embolization. The imaging biomarkers reviewed here provide a practical framework for response evaluation by quantifying gland volume, bladder emptying, and ischemic changes alongside patient-reported outcomes. Among these, CGV and imaging evidence of central gland infarction currently show the strongest associations with clinical outcomes and may represent the biomarkers closest to clinical standardization. For example, recent evidence suggests that infarction extent may be a more durable predictor of clinical success than prostate volume reduction alone. These biomarkers are likely to be most useful when applied within a structured multiparametric framework that integrates baseline characterization, post-procedural assessment, and defined follow-up time points. However, most available data on imaging biomarkers after PAE are derived from single-center retrospective studies or small prospective cohorts with heterogeneous imaging protocols, follow-up intervals, and outcome definitions, limiting direct comparison across studies. Broader clinical adoption will require prospective validation in larger PAE-specific cohorts and consensus on standardized imaging protocols and follow-up timing. As PAE becomes more widely utilized, continued refinement of imaging biomarkers will support a more objective and standardized approach to treatment-response assessment.

## Data Availability

No datasets were generated or analysed during the current study.
